# Myc Oncogene-Induced Genomic Instability: DNA Palindromes in Bursal Lymphomagenesis

**DOI:** 10.1371/journal.pgen.1000132

**Published:** 2008-07-18

**Authors:** Paul E. Neiman, Katrina Elsaesser, Gilbert Loring, Robert Kimmel

**Affiliations:** 1 Divisions of Basic Science, Human Biology and Clinical Research, Fred Hutchinson Cancer Research Center, Seattle, Washington, United States of America; 2 Department of Medicine, University of Washington School of Medicine, Seattle Washington, United States of America; University of California Irvine, United States of America

## Abstract

Genetic instability plays a key role in the formation of naturally occurring cancer. The formation of long DNA palindromes is a rate-limiting step in gene amplification, a common form of tumor-associated genetic instability. Genome-wide analysis of palindrome formation (GAPF) has detected both extensive palindrome formation and gene amplification, beginning early in tumorigenesis, in an experimental Myc-induced model tumor system in the chicken bursa of Fabricius. We determined that GAPF-detected palindromes are abundant and distributed nonrandomly throughout the genome of bursal lymphoma cells, frequently at preexisting short inverted repeats. By combining GAPF with chromatin immunoprecipitation (ChIP), we found a significant association between occupancy of gene-proximal Myc binding sites and the formation of palindromes. Numbers of palindromic loci correlate with increases in both levels of Myc over-expression and ChIP-detected occupancy of Myc binding sites in bursal cells. However, clonal analysis of chick DF-1 fibroblasts suggests that palindrome formation is a stochastic process occurring in individual cells at a small number of loci relative to much larger numbers of susceptible loci in the cell population and that the induction of palindromes is not involved in Myc-induced acute fibroblast transformation. GAPF-detected palindromes at the highly oncogenic *bic/miR-155* locus in all of our preneoplastic and neoplastic bursal samples, but not in DNA from normal and other transformed cell types. This finding indicates very strong selection during bursal lymphomagenesis. Therefore, in addition to providing a platform for gene copy number change, palindromes may alter microRNA genes in a fashion that can contribute to cancer development.

## Author Summary

Genetic instability is a key process in the development of naturally occurring cancer. Gene amplification is one important consequence of underlying oncogenic instability. Long DNA palindrome formation is a rate-limiting early step in gene amplification. The development of a new functional genomic tool called genome-wide analysis of palindrome formation (GAPF) led to the recent discovery of the widespread occurrence of such palindromes in both animal tumor models and human tumor cells. Using a Myc oncogene-induced lymphoma model system, this paper describes clustering of tumor-specific palindromes throughout the genome as well as an association of sites of palindrome formation with both preexisting short inverted DNA repeat sequences and occupied Myc DNA-binding sites. We discovered consistent palindrome formation at a cancer-associated microRNA gene called *bic/miR-155*, beginning at an early stage of tumor development, and without significant further amplification of the locus. Thus, DNA palindromes themselves may directly influence tumorigenesis.

## Introduction

Oncogenic deregulation of the c-*myc* protooncogene was first reported in lymphomas of the chicken bursa of Fabricius resulting from Avian Leukosis Virus (ALV) insertional mutagenesis [1],[2]. Retroviral vector-mediated overexpression of c-*myc* in embryonic bursal precursors induces multi-staged tumorigenesis beginning with preneoplastic transformed follicles (TF) [3],[4] that progress to metastatic B-cell lymphomas. Extensive investigation of deregulated expression of c-Myc more broadly implicates this oncoprotein in a wide variety of animal and human neoplasms [5],[6] as a significant contributor to many of the key processes underlying cancer [7]. These tumor-related phenotypes include genomic instability [8]–[10], which is thought to be essential for the development of most naturally occurring cancers.

Using tools of functional genomic analysis we have recently examined DNA copy number instability during development of Myc-induced bursal lymphomas [11]. For this purpose we employed a 13K chicken cDNA microarray, specifically enriched for chicken immune system and bursal lymphoma ESTs [12], With this tool we carried out array-based comparative genomic hybridization (array-CGH) and detected genome-wide DNA copy number change at many loci in both early TF and end stage lymphomas. Formation of long inverted repeats or palindromes is thought to be a conserved rate-limiting step in eukaryotic gene amplification [13]–[15]. Such palindromes, detected by their ability to form “snap-back” structures in single stranded DNA, have been found in well-characterized tumor-specific amplicons, for example at the c-*myc* locus itself [16]. A functional genomics tool for the genome-wide analysis of palindrome formation (GAPF) recently has been developed and used to detect the widespread presence of palindromes in human cancer [17],[18]. We adapted this technique to interrogate this Myc-induced tumor system [11], and detected extensive palindrome formation in early TF and end-stage lymphomas. The population of loci showing amplification by array CGH was enriched for palindromes detected by GAPF providing strong evidence for genetic instability early in Myc-induced tumorigenesis and further support for the role of palindromes in gene amplification.

In this report we test, in the bursal lymphoma model, the relationship between the non-random formation of palindromes and pre existing short inverted repeats and describe a positive relationship between Myc expression levels and the abundance and genomic distribution of GAPF-detected palindromes. By combining GAPF with chromatin immune precipitation (ChIP) we discovered an association between palindrome formation and the occupancy of nearby Myc binding sites in chromatin, and, finally, report a strong selection for palindrome formation at an oncogenic micro RNA gene, *bic/miR-155*, known to cooperate with *c-myc* in bursal lymphomagenesis [19]–[21].

## Results

### GAPF-Detected Palindromes Are Abundant and Distributed Non-Randomly throughout the Genome of Bursal Lymphoma Cells, Frequently at Pre-Existing Short Inverted Repeats

**Figure 1:**
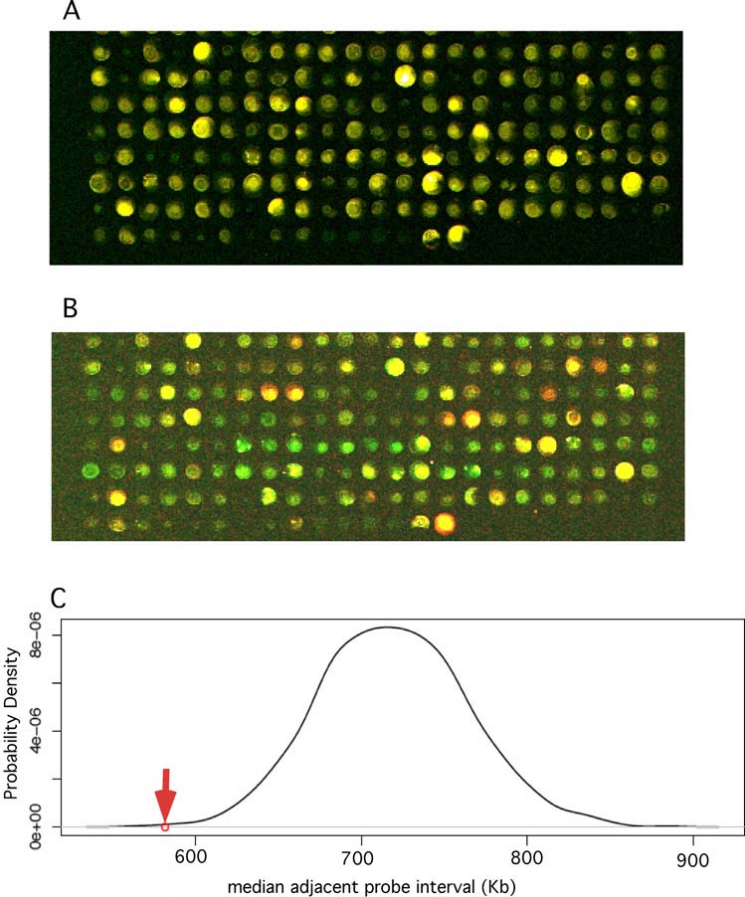
GAPF detects hundreds of apparent palindromes in DNA from DT40 bursal lymphoma cells distributed in a non-random fashion in the genome. (A) Input vs. input controls. About 100 ng of DNA from DT40 clone 8 cells was amplified in two equal samples, and one labeled with Cy 3 (green) and the other with Cy 5 (red) dye, mixed and hybridized to the cDNA micro array (see Materials and Methods). Raw pseudo colored scan data is shown for a section of the array. Yellow colored spots indicate the expected equal representation of the array feature sequence in both DNA sample. (B) Input vs. snap back experiment. One µg of DT40 clone 8 DNA was subjected to the snap-back, S1 nuclease digestion and amplification procedures, labeled with Cy 5 dye, mixed with Cy 3 labeled input DNA and hybridized to the array. The same section of the array as depicted for the input control shows red (Cy-5 pseudo colored) spots representing loci enriched by the snap-back procedure, i.e. presumptive palindromes, and yellow and green spots represent loci which were not enriched (yellow) or depleted (green). (C) Permutation analysis of palindrome positions in DT40 DNA. In order to score as a presumptive palindrome in our GAPF experiment a locus had to have a Cy-5 fluorescent signal strength at least three times background, a Cy-5/Cy-3 fluorescence ratio of at least +2 in the experimental sample and within + or − 0.5 in the input vs. input controls. In seven independent GAPF studies of DT40 clone 8 DNA we detected 643 loci that scored one or more times as palindromes. We tested whether this set of palindromes tended to cluster non-randomly across the genome by comparing the observed median value for the distribution of interval genomic sequence distance between adjacent palindromes (red arrow) to the distribution of median adjacent intervals generated by 1000 permutations of 643 loci along the chicken genome. The genomic pattern of palindrome formation that we observed in this system indicated a distinctly non-random process (P<0.001).

Conditions used for palindrome detection favor rapid formation of duplex fold back structure in denatured DNA followed by nuclease digestion of residual single strands, as described [15],[16] and detailed in Materials and Methods. In our previous GAPF studies we compared palindromes in normal chicken bursal DNA with DNA from preneoplastic and neoplastic bursal tissue, and employed constructs with direct repeats and inverted palindromic repeats as internal negative and positive specificity controls respectively [11],[22]. Using our chicken cDNA microarray GAPF detected very few palindromes in DNA from normal bursa. In view of this result, and In order to combine GAPF with other techniques for these studies, we modified our GAPF technique to compare bursal tumor cell DNA before and after the “snap-back” procedure, an approach employed successfully by other investigators [15],[17]. We employed this form of GAPF on DNA from a clone (clone 8) of DT40 cells, a bursal lymphoma-derived cell line [23] with a typical genomic complement of amplified and palindromic loci [24]. In repeated experiments we consistently detected hundreds of loci containing presumptive palindromes in DT40 clone 8 cells. We carried out a permutation analysis that compared the distribution of genomic sequence intervals separating 643 putative palindromes in DT-40 DNA with a 1000-fold reiterated random distribution of 643 loci in the genome. This study, described in Figure 1, indicated that the distribution of palindromes was distinctly non-random, and suggested the presence of sites or regions of preference for palindrome formation in this system.

**Figure 2:**
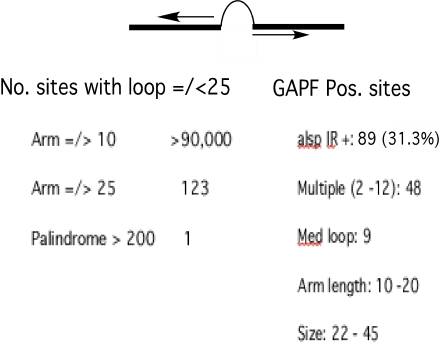
A significant portion of GAPF-detected palindromes in DT40 cells occurs at sites of short IRs in chicken DNA. A generalized short IR is depicted with the complementary “arms” as a thick black line separated by a non-complementary “loop” sequence. The column on the left lists numbers of IRs with loops of 25 nucleotide base pairs or less. These numbers drop dramatically as arm length increases from a minimum of10 base pairs to a minimum of 25 base pairs. Only one palindrome was detected with an arm length greater than 200 base pairs. On the right are listed the relevant features of 89 IRs with loops of up to 25 base pairs found within 1 kb of 284 apparent palindromes that were detected in 50% or more of GAPF studies of DT40 cells. Most of these GAPF positive sites had multiple nearby IRs with loop sizes, arm lengths and overall sizes as shown.

In Tetrahymena, yeast and mammalian cells short inverted repeats (IRs), adjacent to sites of double strand DNA cleavage, mediate long palindrome formation and subsequent gene amplification [13]–[15]. Sequences encoding such IRs, known to be plentiful in mammalian DNA [25], have been postulated to provide substrates for palindrome formation [15]. In order to evaluate this possibility we scanned the chicken genomic sequence for IRs similar to those known to stimulate palindrome formation, and looked for an association with sites of palindrome formation detected in this study. Figure 2 summarizes the results of a chicken genomic scan for short IRs and the overlap of these sites with GAPF detected palindromes in DT40 cells. If we set an arbitrary size range of up to 20 nucleotides for the non complementary loop separating the palindromic arms of the IRs, the number of IRs detected varied from about 104,000 to only 123 as the perfectly paired arm length increased from a minimum 10 to a minimum of 20 nucleotides. Only one palindrome with an over-all length of greater than 200 nucleotides was detected. Figure S1 provides a more complete survey of the frequency of IRs in the chicken genome with a range of perfect complementary arm lengths and a loop size of up to 25 bp.

As summarized in Figure 2, 31.3 percent of the 284 GAPF positive sites that were consistently detected in replicate studies with DT40 DNA contained IRs within 1 Kb up or downstream of the center of the EST sequence for the GAPF positive feature on the array. The hyper geometric probability of a chance association of this magnitude is about 1.8×10^−5^. A majority of these sites contained multiple IRs with a median loop size of 9 nucleotides, arm lengths between 10 and 20 nucleotides and a range of overall length between 22 and 45 nucleotides. These findings represent a minimum correspondence between long palindromes and preexisting short IRs because IR loop sizes could easily be larger, and we don't have complete sequences for cDNAs on the array.

### Occupation of Myc Binding Sites in Chromatin Overlaps with Sites of Formation of Palindromes and the Numbers of Both Types of Sites Increase with Myc Overexpression

In order to test for a relationship between Myc binding and palindrome formation we employed a chromatin immune precipitation assay for genome-wide analysis of Myc binding sites (ChIP on chip analysis), and combined this approach with GAPF. We began by analyzing chromatin from DT40 bursal lymphoma cells, which express high levels of cMyc and then examined v-Rel transformed chicken B cell lines in which levels of Myc expression varied >50 fold. The results of the ChIP on chip

**Figure 3:**
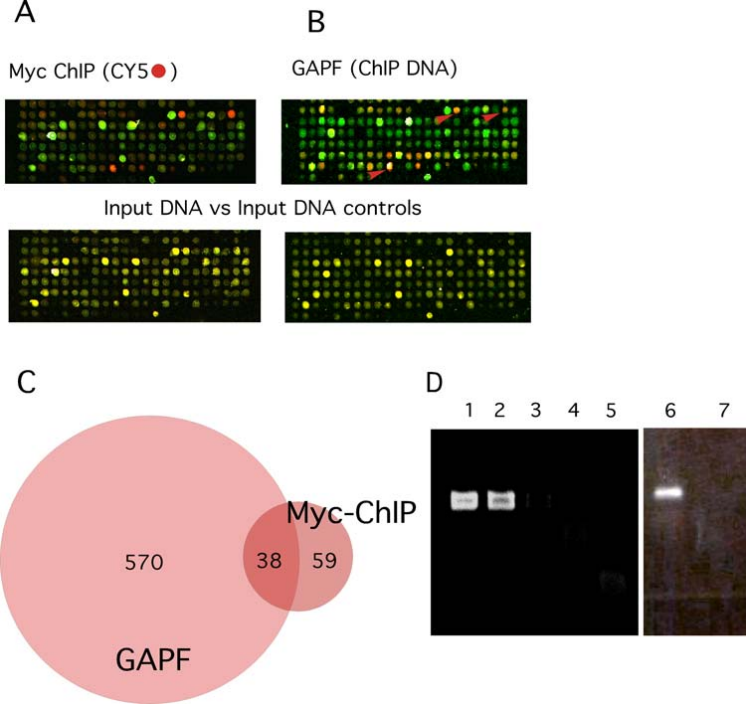
Combined ChIP and GAPF studies of DT40 bursal lymphoma cells indicates the presence of palindromes near Myc binding sites. (A) DT40 clone 8 cell chromatin was cross linked with formaldehyde, fragmented by sonication and precipitated with a monoclonal antibody, 9E11,which recognizes chicken cMyc. DNA was recovered from the immunoprecipitates, amplified by ligation-mediated polymerase chain reaction (PCR),labeled with Cy-5, mixed with Cy-3 labeled input DT40 clone 8 DNA, and hybridized to the microarray essentially as described [48] and detailed in Materials and Methods. Pseudo-colored scans of a section of the array show loci in red, which were enriched by 9E10 immunoprecipitation (presumptive Myc binding sites), and loci depleted by immunoprecipitation in green. Control DNA input v*s*. input studies are shown on the lower panel for comparison. (B) GAPF was carried out on DNA from the same DT40 clone 8 cell culture used for the ChIP analysis. On the same section of the array Cy5-labeled (red) spots represent palindromic loci enriched by the snap-back protocol, and arrows indicate correspondence with ChIP positive loci. Input *vs.* input controls for the GAPF studies a shown for comparison on the lower panels. (C) The Venn diagram indicates overlap between ChIP and GAPF positive loci. By hypergeometric distribution the probability of a chance overlap of this extent was very low (P<9.22×10^−22^). (D) We used gene-specific ChIP to validate representative ChIP positive loci detected on the array. Five loci that scored strongly by both GAPF and ChIP on chip analyses were selected. Data is shown from two examples, Metaxin Binding Protein (MBP) and the non-coding RNA Bic, which like the others, have presumptive high affinity Myc binding sites (CACGTG) just upstream of their transcriptional start sites. Primers spanning these binding sites were used in PCR to amplify the MBP sites, lanes 1 to 5, or the *bic* site, lanes 6 and 7. PCR products of the correct size for the MBP site, lane 2, and from the *bic* site, lane 6, are shown for chromatin precipitated with the 9E11 Myc antibody, and for a DT40 total DNA positive control for the MBP site, lane 1. In contrast no PCR product from either site was detected, lanes 3 and 7, with a control antibody, 9E10, which recognizes mammalian but not chicken cMyc, or with an immunoglobulin isotype control, lane 4, or a no DNA reagent only control, lane 5.

**Figure 4:**
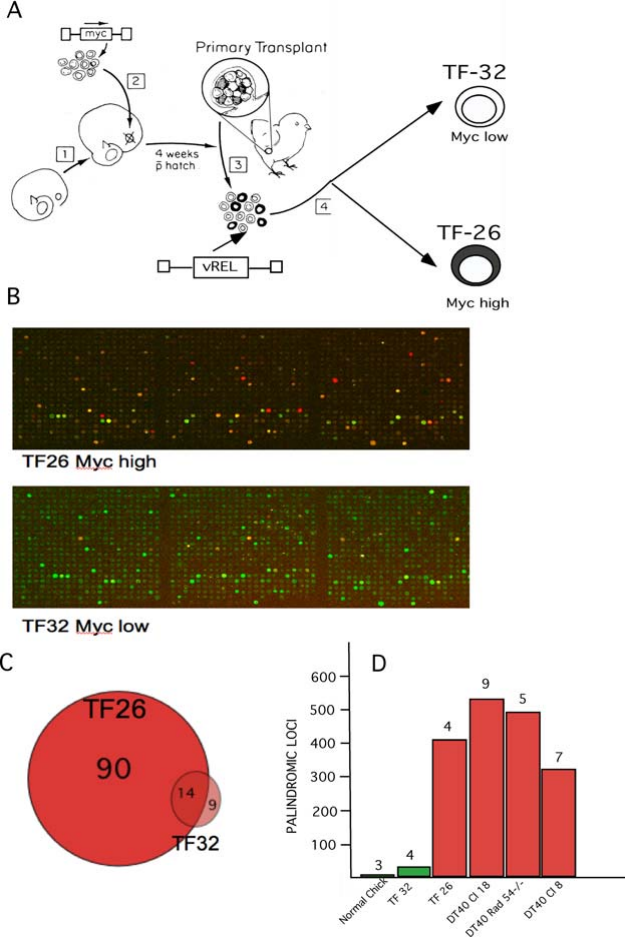
Analysis of transformed B-cells with high and low levels of Myc. (A) This chart shows the protocol for generating high and low Myc expressing bursal cell lines with vRel. Embryonic bursal cell populations were infected with a defective Myc oncogene-transducing retroviral vector LvMycSN [27] and used to reconstitute ablated bursal follicles in a bursal transplantation experiment [3],[4]. Reconstituted bursas contained mixtures of normal follicles and Myc over-expressing TF corresponding to successfully Myc-transduced bursal stem cells (depicted as black). Mixed normal and TF cell populations, from mixed normal follicles and TF, were then infected with Reticuloendotheliosis Virus, strain T, a vRel transducing retrovirus that rapidly transforms all chicken B-cells yielding clonal cell lines [26]. TF 32 is such a line of vRel transformed normal bursal cells and TF 26 a line of vRel transformed TF cells with a ∼50 fold increase in Myc expression [27]. (B) A section of the microarray from a ChIP on chip experiment with chromatin from high Myc TF 26 cells (upper panel) shows higher numbers of occupied Myc binding sites than observed on the same section of the array for low Myc TF 32 cells (lower panel). (C) The Venn diagram summarizes totals of occupied Myc binding sites in TF 26 and 32 cells. (D) This chart summarizes total numbers of palindromes consistently detected by GAPF in low Myc expressing normal bursal cells and TF 32 cells (green columns) in contrast with high Myc expressing (red columns) TF 26 cells and three different clones of DT40 bursal lymphoma cells, clone 8, clone18 parental and rad 54 -/- cells derived from clone 18. The numbers of individual GAPF microarray experiments used to derive totals of recurrently detected palindromes (>50% of experiments) are given above each column. Targeted deletion of Rad 54 from DT40 clone 18 was carried out and characterized by J-M Buerstedde and colleagues [28].

Analysis of DT40 bursal lymphoma cells is detailed in Figure 3 and indicates a strong association between some Myc binding sites and some loci of palindrome formation, but does not prove a causal relationship. One prediction of that possibility would be that increasing levels of Myc should increase both the numbers of occupied Myc binding sites and the numbers of palindromes detected by our functional genomic assays. As described in Figure 4 we tested this possibility in chicken B cells transformed by the NF Kappa B related viral oncogene vRel[26]. This oncogene can be used to acutely transform both normal bursal B-cells expressing low levels of cMyc and pre-neoplastic bursal TF cells expressing high levels of cMyc [27]. Figure 4A charts a protocol for generating sister vRel transformed bursal cell lines expressing either high or low levels of cMyc [27]. Figures 4B and 4C demonstrate a marked increase in numbers of occupied Myc binding sites detected by ChIP on chip assays in the high cMyc expressing cells compared to low Myc expressing vRel transformed bursal B-cells. Figure 4D demonstrates the increased numbers of GAPF-detected palindromes in the high Myc vRel transformants and compares these totals detected by GAPF in several DT40 clonal cell lines as well as normal bursa. As also shown in Figure 4D the numbers of GAPF-detected palindromes were not reduced in DT40 cells in which homologous recombination had been reduced by targeted deletion of RAD-54 [28].

These results suggest a quantitative relationship between the level of deregulated Myc overexpression and palindrome formation. Transformation by vRel in the presence of normally low levels of cMyc in bursal cells produced only minimal increases in palindromes suggesting that neither B-cell transformation itself, nor all types of transcription factor activity itself, could induce large numbers of palindromes. Finally, DT40 cells are unusual among vertebrate cell lines in their exceptional capacity for homologous recombination [29], which might contribute to the increased palindrome formation we have observed. This property has been useful for targeted gene deletion studies. Homozygous deletion of Rad 54 in DT40 clone 18 cells has been shown markedly to reduce homologous recombination toward normal levels [28], but did not appear to affect palindrome formation in our experiments (Figure 3 D).

### Clonal Analysis of Immortalized Untransformed and Myc-Transformed DF-1 Chick Fibroblasts Reveals that Palindrome Formation Is a Stochastic Process in Individual Cells Occurring at a Relatively Small Number of Sites within a Larger Population of Susceptible Sites

Cultured primary chick embryo fibroblasts express low, barely detectable levels of c-myc RNA and protein. DF-1 is an immortalized morphologically normal chick embryo fibroblast cell line that can be transformed acutely by the oncogenes of a number of avian retroviruses [31]. We carried out GAPF studies with DNA from mass DF-1 cultures and new clones of uninfected DF-1. Low numbers of palindromes were detected in DNA from mass cultures of DF-1 cells. In contrast, when examined by GAPF, DNA from 10 individual DF-1 clones contained a variable, but on average about an 8-fold, increase in total palindromes compared to mass DF-1 cultures. These results in DF-1 cells suggested that formation of palindromes is a stochastic process, and the sequence target size is relatively large compared to the number formed in any one cell, so that patterns are more readily detected in single cell clones than in mass cultures where contributions from individual cells may be diluted below the sensitivity of the assay. DF-1 cells are immortal, and have been cultivated for many years. Therefore we could not distinguish between palindromes generated *in-vivo* before establishment and those developing, perhaps quite slowly, after establishment in culture.

We then rapidly transformed DF-1 cells by high multiplicity infection with a Myc-transducing retrovirus, HB1 [32]. GAPF studies of Myc-transformed DF-1mass cultures and a series of 10 transformed DF-1 clones initiated two weeks after infection and transformation also revealed palindromes principally, in transformed cell clones rather than mass cultures. As shown in Figure S2 the numbers of palindromes detected by GAPF were not altered by acute vMyc transformation of DF-1 in either mass culture or individual clones. Therefore, and In contrast to our observations in the multistage development of bursal lymphomas, palindrome formation is unlikely to play an essential role in acute Myc transformation of DF-1 fibroblasts

### Palindromes Formed at *bic/miR-155* Loci Are Strongly Selected during Tumorigenesis

*Bic* is a non-coding RNA gene discovered as a site of ALV insertional mutagenesis (in addition to activating insertions at c-*myc* ) selected during tumor development in a large proportion of ALV-induced bursal lymphomas [19],[20]. Subsequently *bic* was found to encode a highly conserved micro RNA, miR-155, shown to be essential for normal B-cell development [33], to be lymphomagenic when over expressed in transgenic mice [34], and to be over expressed in several forms of human B-cell malignancies [35]–[37]. Although enforced *bic* overexpression was shown to accelerate the development of ALV induced bursal lymphomas [21], *bic* was not activated by retroviral insertion in all ALV-induced bursal lymphomas [19] or in derived cell lines like DT 40 [24]. Furthermore, in many experiments with bursal lymphomas induced by Myc-transducing defective retroviral constructs, where ALV insertional mutagenesis does not occur, we did not detect increases in spliced *bic* RNA [11]. We did, however, detect palindrome formation at *bic*. In GAPF studies from all of our analyses of preneoplastic TF (three studies), bursal lymphomas (three experiments ) and multiple studies of derivative high Myc expressing transformed B- cell lines such as DT 40 or TF 26 (Figure 3D). In contrast, none of the three GAPF studies of DNA from normal red blood cells or normal bursa, and four studies of low Myc TF 32 cells (vRel transformed normal bursal cells) detected palindromes at *bic*. Our twelve GAPF studies of untransformed DF-1 mass cultures and clones, and Myc-transformed DF-1 cultures and clones, also failed to detect palindromes at *bic*. Thus a high level of deregulated Myc oncogene expression was not sufficient by itself to result in a high frequency of palindrome formation at *bic*. The specific 1 to 1 correlation of palindrome formation at *bic* with all stages of bursal lymphomagenesis (preneoplastic TF, metastatic lymphoma and derivative established cell lines) indicates a strong selection in the development of these tumors for this otherwise uncommon change at the *bic* locus, and indicates a mechanistic alternative to retroviral insertional mutagenesis for alteration of *bic/miR-155*.

**Figure 5:**
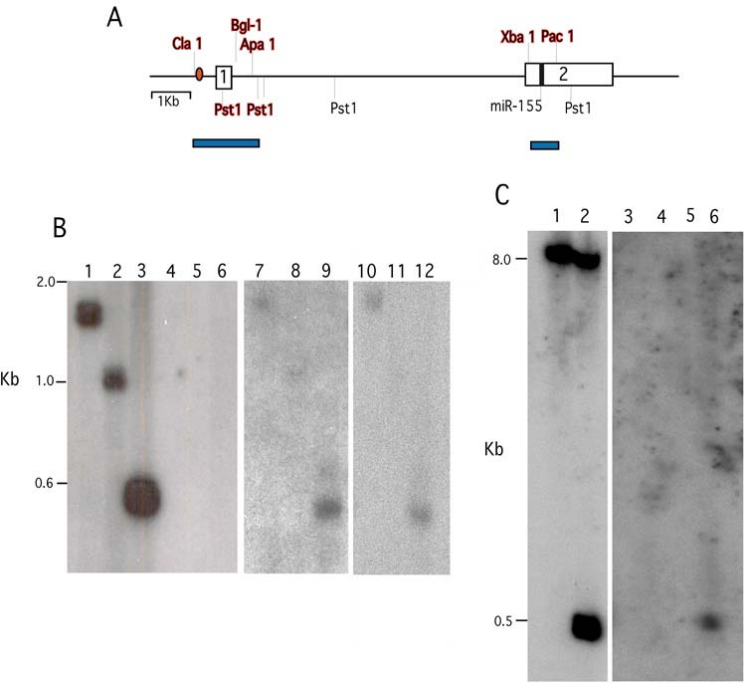
Southern blot-hybridization analysis of the palindromic region near the bic locus. (A) This chart maps the region around *bic* that includes the cDNA probe (exons 1 and 2, white boxes) used in GAPF studies. Positions of the Myc binding site (red oval), exons 1 and 2, the position of miR-155 coding sequence (black box) and relevant restriction cut sites are indicated. Restriction sites preserved after the snap-back procedure, S1 digestion and amplification, as detected by blot hybridization, are indicated in red bold face and mapped with blue bars. (B) Blot hybridization results for normal bursal DNA (lanes 1 to 6), DT-40 DNA (lanes 7 to 9), and bursal lymphoma DNA (lanes 10 to 12) before snap-back and S1 digestion (lanes 1 to 3), and after this procedure (lanes 4 to 12). Both input and S1 resistant DNA was digested with restriction enzymes; Cla 1+Apa 1 (lanes 1, 4, 7 and 10), Cla 1+Bgl 1 (lanes 2, 5, 8 and 11) or Pst1 (lanes 3, 6, 9 and 12). A ^32^P-label *bic* exon 1 probe was used to analyze the blot. (C) Normal bursal DNA before snap-back (lanes 1and 2) and DNA after snap back, S1 digestion and amplification from normal bursa (lanes 3 and 4) and bursal lymphoma ( lanes 5 and 6) were digested with Pac-1 (lanes 1, 3 and 5) or Pac-1 and Xba-1 (lanes 2, 4 and 6). Blot-hybridization was carried out with a ^32^P-labeled *bic* exon 2 probe.

In order to validate the GAPF results for the *bic* locus, and gain an initial insight into the structure of palindromes formed near *bic*, we carried out blot-hybridization studies of DNA from normal bursa, DT40 cells and bursal lymphoma from an *in-vivo* tumor before and after the snap-back procedure and digestion with S1 nuclease. Figure 5 depicts a chart of the genomic region around bic including the positions of a Myc-binding E box (CACGTG) exons 1 and 2, the location of miR-155, key restriction endonuclease sites and the results of Southern blot hybridization analysis. These results indicate that the region around *bic* exon 1 and a segment of exon 2 in DT40 and bursal tumor DNA, but not in normal chicken DNA, retains an S1 resistant duplex character after the snapback procedure, consistent with the GAPF results. It is difficult to assess the significance of the relatively weaker blot-hybridization signal of the S1 resistant *bic* DNA recovered from tumor cells, in comparison to that from the pre-snap back normal control DNA. Influences on signal strength arising from the amplification procedure, S1 digestion of one normal *bic* allele, and/or structural heterogeneity at the *bic* locus in DNA of the tumor cell population may all play a role in diminishing the signal. In any case we conclude that the S1 resistant segments detected are included in a complementary paired region of a palindrome at the *bic* locus of DT40 and bursal tumor cells that is not present in normal chicken DNA.

## Discussion

We concluded that the deregulated overexpression of Myc oncoproteins in the bursa results in the widespread accumulation of long DNA palindromes not normally present in chicken bursal B-cells. This response did not extend to constitutive overexpression of another oncogenic transcription factor, vRel, nor was it a consistent general property of the transformed state of B-cells or fibroblasts. Analysis of the genomic distribution of these structures with respect to clustering, the presence of pre-existing IRs and occupied high affinity Myc binding site indicated the existence of preferred sites for palindrome formation. However, the sub genomic target size for induction of palindromes still appears to be quite large in comparison to the numbers of palindromic sites found in individual tumors. As with our previous study of gene amplification in this system [11] there was no unique signature of affected genes detected with the very important exception of the Bic/miR-155 locus. These observations, at the DNA level, are reminiscent of the historic detection of highly variable cytogenetic instability accompanying clonal evolution early in tumorigenesis leading to the selection of single essential oncogenic loci at sites of chromosomal rearrangement [38].

The GAPF studies of clones of untransformed DF-1 fibroblasts, in comparison with studies of parental mass cultures, suggests some important caveats in drawing conclusions about DNA palindromes in clonal neoplasms. The known genotoxic effects of long palindromes may dictate the instability of newly formed DNA palindromes in both germ line and normal somatic cells [39] and explain the relatively low numbers of palindromes detected In normal tissue DNA. Clones of morphologically normal DF-1 fibroblasts, however, contained significantly increased numbers of GAPF detected palindromes, which could have accumulated in single cells either during somatic life and/or as a result of immortalization and extended growth in tissue culture after establishment. Comparison of clonal tumor DNA with normal somatic tissue DNA for differences in palindromes, therefore, needs to be interpreted with some caution.

The results of the effects of Myc overexpression in this set of experiments, however, are not explained simply by the clonal nature of tumor cells. For example low Myc expressing TF 32 is a clonal cell line derived from normal bursal cells with many fewer palindromes than the clonal TF 26 line derived from Myc-transformed TF cells. Furthermore non-clonal Myc-induced preneoplastic transformed bursal follicle populations show marked increases in palindrome formation over normal bursal DNA[11]. Therefore genomic instability detected by GAPF in DF-1 clones, but not in either mass transformed cell cultures or transformed DF-1 clones, suggests that this process is not involved in acute single step Myc-induced fibroblast transformation. In contrast multistage development of bursal lymphomas may rely on this type of genomic instability established early in the preneoplastic phases of this disease. This conclusion is strongly supported by the consistent selection of palindromes at the highly oncogenic *bic/microiRNA-155* locus during bursal lymphomagenesis. Why bursal target cells apparently differ from DF-1 fibroblasts with respect to palindrome formation and transformation in response to Myc is unknown. These bursal cells are exquisitely sensitive to radiation and other genotoxic agents [3]. Therefore double strand DNA breaks, the initiating lesions in palindrome formation, may be formed and/or repaired in a manner favoring establishment of palindromes in these cells.

It would be desirable to know the molecular consequences of this change. Palindrome formation at *bic* was not accompanied by gene amplification that was detectable by array CGH in our experiments [11]. Furthermore, and in contrast to ALV-induced lymphomagenesis, in this experimental model we have not detected any change in the low levels of spliced *bic* RNA during tumor development. We have carried out PCR-based assays (QuantiMir RT kit, SBI Systems Bioscience, Mountain View, CA) of miR-155 in whole normal embryonic and post hatching bursa as well as from preneoplastic TF and bursal lymphomas(not shown). As might be anticipated from the complex regulatory role of this micro RNA in vertebrate B-cell development [33], there was universal expression in all these tissues, but no simple pattern of accumulation correlating with neoplastic change. We do not know, however, the normal level of miR-155 expression in target normal bursal stem cells, which is the critical factor for comparison with expression in Myc-induced neoplastic development. We are left with the speculation that there may be a regulatory alteration in expression, in critical target cells, of this highly conserved pleiotropic micro RNA, which will require further study. In any case these results suggest that, in addition to providing a platform for gene amplification, palindrome formation can be a novel mechanism for oncogenic alteration of micro RNAs.

There are a number of known mechanisms for Myc-induced genomic instability that could contribute to the formation of palindromes that we have detected in these studies. In principle any process that produces DNA double strand breaks could initiate the formation of long palindromes, for example at short IRs. As mentioned, cytogenetic abnormalities associated with Myc overexpression, which are known to result from such breaks, have been described in mammalian cell lines [8],[9] and in DT40 cells [40]. Recently, Gp1bα, a subunit of the vonWillebrand factor receptor complex and a transcriptional target of Myc, has been implicated in a pathway from Myc-driven overexpression to tetraploidy and DNA strand breaks [41],[42]. Additionally, c-Myc has been shown to play a direct role in DNA replication by localizing at early sites of DNA synthesis. When over expressed, c-Myc drives increased replication origin activity and subsequent DNA damage [43]. Either or both of these of mechanisms in principle could contribute to the widespread formation of palindromes that we have observed. Neither mechanism, however, either requires or explains the striking association that we have detected on a cDNA micro array between sites of Myc DNA binding at or near exons and sites of palindrome formation. The association of course could reflect an unknown structural feature shared between sites of Myc binding and palindrome formation with no mechanistic connection to genomic instability. Alternatively it could also reflect an unknown cis-acting effect of Myc binding site occupancy. For example, with respect to DNA strand cleavage, activation of transcription by a number of DNA binding proteins produces transient DNA double stand breaks near the start of transcription as a normal physiological event [44]. As shown in this study pathologically deregulated Myc overexpression results in a marked increase in occupied Myc binding sites where abnormal activation events might, in a stochastic fashion, perturb the normally rapid repair of such breaks and thereby lead to palindrome formation. While at this point clearly speculative, such a mechanism fits well with our observations in this study.

## Materials and Methods

### Preneoplastic Myc-Transformed Follicles, Bursal Lymphomas, and Derivative Cell Lines and Immortalized DF-1 Chick Embryo Fibroblasts

The bursal transplantation model, employing defective c-*myc* transducing retroviral vectors to generate preneoplastic transformed bursal follicles (TF), and metastatic bursal lymphomas, has been described previously [11]. Generation and culture of TF 26 vRel- transformed TF, and TF 32 vRel-transformed normal bursal cell lines is outlined in Figure 4 and was described previously [27]. DT40 bursal lymphoma cells and DF-1 immortalized chick embryo fibroblasts were maintained as described respectively [23],[31]. DNA for functional genomic analyses was prepared from tissues and cell lines as described [45].

These experiments employed a 13K chicken cDNA microarray [12] used previously for expression profiling, comparative genome hybridization analysis of gene copy number and initial GAPF studies in this system [11], As mentioned in the text we modified our original GAPF protocol [11],[22] essentially as described previously [15]. In brief, one µg of DNA was denatured in 100 µl of 10 mM Tris, 100 mM NaCl at 100° for seven minutes, cooled rapidly on ice for 2 minutes to form fold back duplexes, and single stranded DNA was digested for 60 minutes with 1.6 units/µl of S1 nuclease in 1×New England Biolabs buffer 2 plus 20 mM NaCl at 37°. Duplex “snap back” DNA was then amplified using a whole genome amplification kit (Repli-g, Qiagen, Valencia, CA), reduced in size by digestion with Alu-1 plus Rsa-1, labeled with Cy-5, mixed with equal amounts of amplified, digested and Cy-3 labeled input DNA and hybridized to the microarray in the presence of 0.7 µg/µl of normal chicken Cot-1 DNA. Hybridization, scanning and spot intensity signal filtration (Genepix pro and GD filter software) parameters were as described, and spot-level Cy-5/Cy-3 ratios log_2_ transformed as before.

### Scan of Chicken Genome for Short Inverted Repeats

Naturally-occurring DNA palindromes in the Gallus gallus genome were detected by scanning the UCSC chicken genome sequence database, May 2006, version 2.1 draft assembly [46],[47]. DNA palindromes were defined as inverted repeat (i.e. *cis*-wise, self-complementary) sequences having arms of specified minimum length that are connected by loops of specified maximum length. Individual chromosomal maps were searched directly using an R-script [48] routine (see code in Protocol S1) that iteratively identified all sequences meeting user-specified arm- and loop-length criteria For examples see Figure 2. Culling shorter sequences having the same loop retained the longest unique palindromes. Each palindrome center was defined as the midpoint of its loop.

### Myc Chromatin Immune Precipitation (ChIP) and ChIP on Chip Analysis

These studies followed, essentially, the procedure of Ren et. al. [49]. In brief, formaldehyde cross-linked and sonicated chromatin fragments were prepared from DT40, TF-26 and TF-32 cells and immunoprecipitated with either 9E11 Myc monoclonal antibody or control 9E10 monoclonal antibody and IgG-2A isotype control immunoglobulin (Abcam, Cambridge, MA). Formaldehyde cross-linking was then reversed and DNA recovered. Immunoprecipitated DNA was amplified by ligation-mediated PCR, and for genome-wide localization, labeled with CY-5 mixed with sonicated Cy-3 labeled input DNA and hybridized to the microarray. Signals were detected and processed in a fashion identical to that employed for GAPF. For gene locus-specific Myc ChiP assays, amplified DNA from immunoprecipitates with chicken Myc antibody and control antibody was subjected to gene-specific quantitative PCR using primers spanning apparent high affinity Myc binding sites, CACGTG, near transcription start sites. For Metaxin-BP the primer pairs were, forward: CTGAGAAGTGAAGCTGCAGTCCTG, reverse: GATTACTCACCCG AGGTATGGCTC; for *bic* they were, forward: GCTGAGGTGCTCCAGTGGCAG, reverse: CTAGTCTTCTCTTTGTTGCAGGTC. PCR limit products were evaluated by electrophoresis on 3% agarose gels.

### Southern Blot Hybridization

Ten µg of input normal chicken DNA was digested with restriction endonucleases pst1, cla 1+apa 1 or cla 1+bgl 1. One µg of normal and DT40 DNA was subjected to the same procedure as for the GAPF studies including rapid denaturation - renaturation, digestion with S1 nuclease and amplification. Ten µg of amplified snap-back normal and DT40 DNA was then digested with the same sets of restriction endonucleases. Blot-hybridization was carried out on 1% agarose gels and evaluated with the same *bic* cDNA probe used on the microarray.

## Supporting Information

Figure S1 Frequency of IRs in the chicken genome with a loop length up to 25 nucleotides.
(0.38 MB TIF)

Figure S2 Clonal analysis of DNA palindromes in DF-1 fibroblasts before and after Myc transformation. Total numbers of palindromes detected by GAPF in immortal DF-1 fibroblasts in mass culture and multiple fresh clones before and two weeks after transformation by infection with HB1. The bars represent standard errors of the mean.
(0.18 MB TIF)

Protocol S1 Supplemental programming information. Code for R script for genomic scans of user-defined palindromes.
(0.04 MB DOC)
