# Supplementary figures and images for: Myc Oncogene-Induced Genomic Instability: DNA Palindromes in Bursal Lymphomagenesis
Source: PLoS Genet. 2008 Jul 18;4(7):e1000132. doi: 10.1371/journal.pgen.1000132 (PMC2444050; doi:10.1371/journal.pgen.1000132)

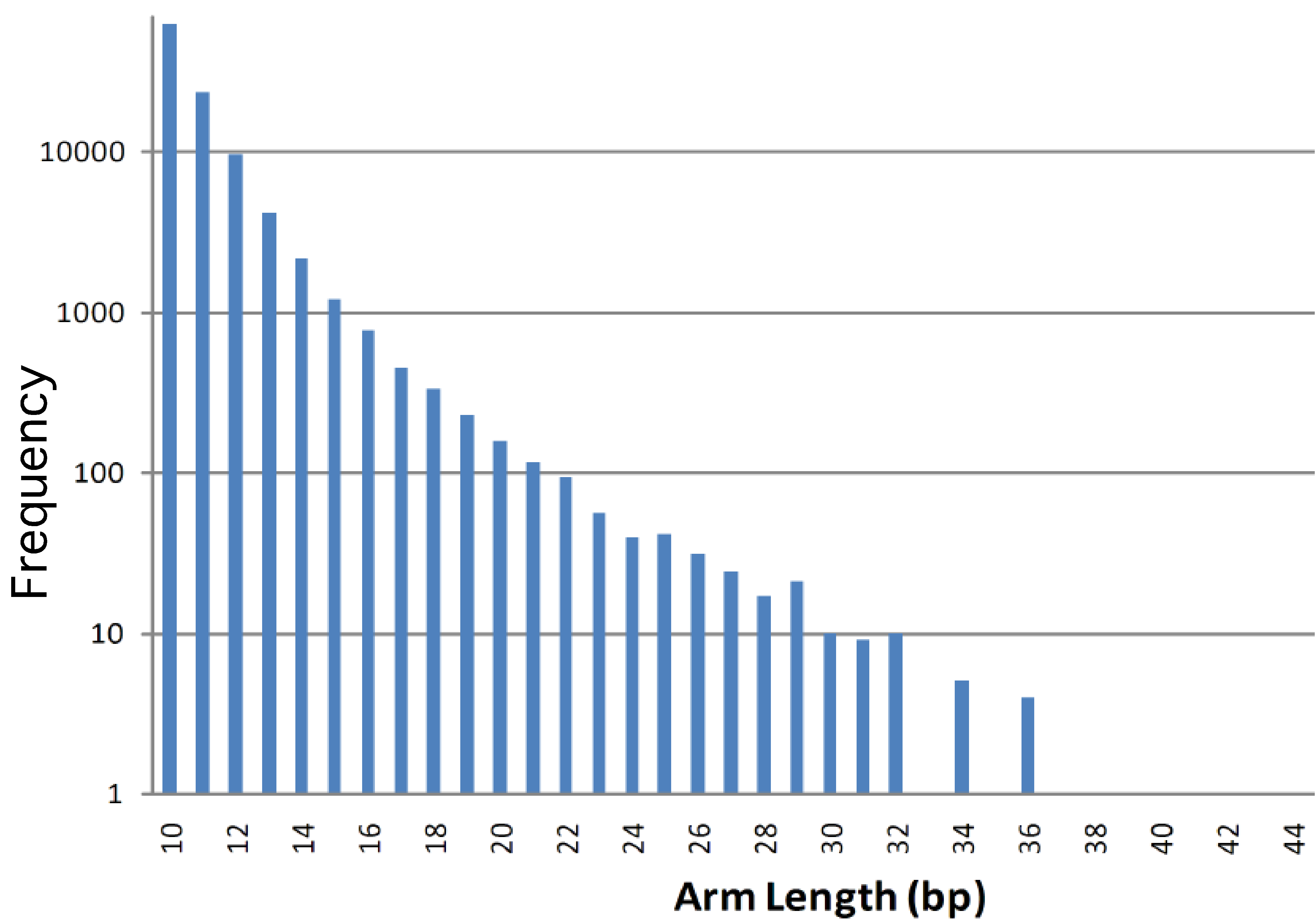

Supplement: Figure S1 — Frequency of IRs in the chicken genome with a loop length up to 25 nucleotides. (0.38 MB TIF) [file pgen.1000132.s001.tif]

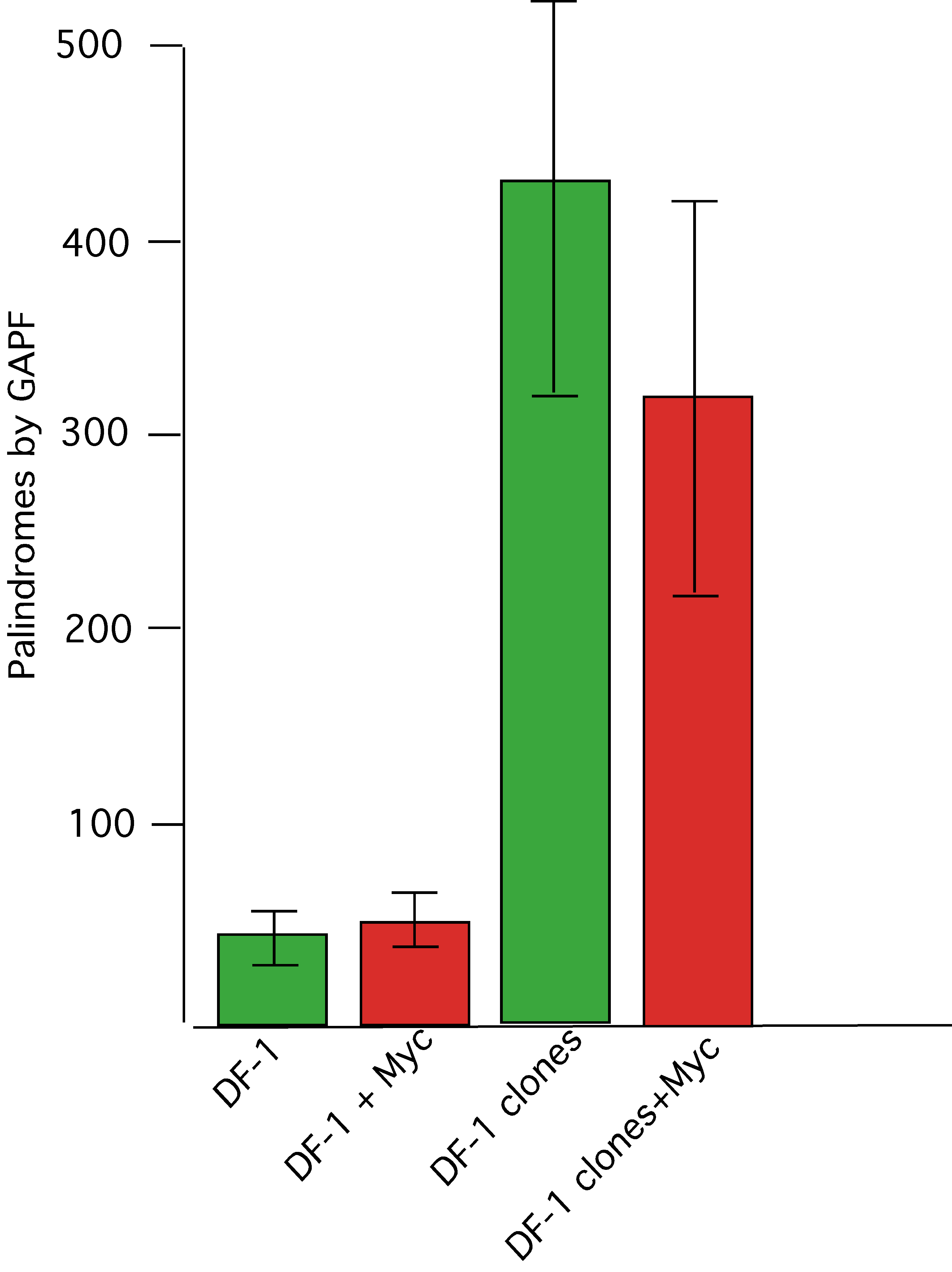

Supplement: Figure S2 — Clonal analysis of DNA palindromes in DF-1 fibroblasts before and after Myc transformation. Total numbers of palindromes detected by GAPF in immortal DF-1 fibroblasts in mass culture and multiple fresh clones before and two weeks after transformation by infection with HB1. The bars represent standard errors of the mean. (0.18 MB TIF) [file pgen.1000132.s002.tif]
