# Supplementary material for: Myc Oncogene-Induced Genomic Instability: DNA Palindromes in Bursal Lymphomagenesis
Source: PLoS Genet. 2008 Jul 18;4(7):e1000132. doi: 10.1371/journal.pgen.1000132 (PMC2444050; doi:10.1371/journal.pgen.1000132)
Supplement: Protocol S1 — Supplemental programming information. Code for R script for genomic scans of user-defined palindromes. (0.04 MB DOC) [file pgen.1000132.s003.doc]

### R SCRIPT FOR FINDING 2-ARM PALIDROMES IN CHICKEN GENOME

### Last modified: Jan 11, 2008

### Author: H. Pages (hpages@fhcrc.org)

SEQNAMES <- c("chr1", "chr2", "chr3", "chr4", "chr5", "chr6", "chr7", "chr8",

"chr9", "chr10", "chr11", "chr12", "chr13", "chr14", "chr15",

"chr16", "chr17", "chr18", "chr19", "chr20", "chr21", "chr22",

"chr23", "chr24", "chr25", "chr26", "chr27", "chr28", "chr32",

"chrM", "chrW", "chrZ", "chrE64", "chrE22C19W28_E50C23")

OUTFILE <- "chickenpals.txt"

MIN.ARMLENGTH <- 25

MAX.LOOPLENGTH <- 200

### ----------------------- end of GLOBAL PARAMETERS ------------------------

library(Biostrings)

library(BSgenome.Ggallus.UCSC.galGal3)

writePalHeader <- function(file="")

{

cat("seqname", "start", "end", "length", "armlength", "looplength", "seq",

file=file, sep="\t")

cat("\n", file=file, append=TRUE)

}

writePals <- function(seqname, pals, file="")

{

for (i in seq_len(length(pals))) {

pal <- pals[[i]]

L <- length(pal)

armlength <- complementedPalindromeArmLength(pal)

if (armlength == L) {

looplength <- 0

armlength <- armlength %/% 2

} else {

looplength <- L - 2 * armlength

}

cat(seqname, start(pals)[i], end(pals)[i], width(pals)[i],

armlength, looplength, as.character(pal),

file=file, sep="\t", append=TRUE)

cat("\n", file=file, append=TRUE)

}

}

palAnalysis <- function(outfile)

{

seqnames_in1string <- paste(SEQNAMES, collapse=", ")

cat("Target: ", providerVersion(Ggallus),

" chromosomes ", seqnames_in1string, "\n", sep="")

if (outfile != "")

writePalHeader(file=outfile)

for (seqname in SEQNAMES) {

subject <- mask(Ggallus[[seqname]], "N")

cat(">>> Finding palindromes in chromosome ", seqname, " ...\n", sep="")

pals <- findComplementedPalindromes(subject,

min.armlength=MIN.ARMLENGTH,

max.looplength=MAX.LOOPLENGTH,

writePals(seqname, pals, file=outfile)

cat(">>> DONE\n")

unload(Ggallus, seqname)

}

}

palAnalysis(OUTFILE)
